# Adie's Tonic Pupil in Systemic Sclerosis: A Rare Association

**DOI:** 10.1155/2015/491795

**Published:** 2015-08-27

**Authors:** Anusha Venkataraman, Bijnya Birajita Panda, Chandrasekhar Sirka

**Affiliations:** ^1^Department of Ophthalmology, All India Institute of Medical Sciences, Bhubaneswar 751019, India; ^2^Department of Dermatology, All India Institute of Medical Sciences, Bhubaneswar 751019, India

## Abstract

We report a rare association of Adie's tonic pupil in a patient with systemic sclerosis who was otherwise systemically stable. This paper is an effort to unravel whether the tonic pupil and systemic sclerosis are an association by chance (which may be the case) or systemic sclerosis is the source of the tonic pupil.

## 1. Introduction

Adie's tonic pupil has a prevalence of 2/1000 cases and is usually a unilateral condition reported in women with a mean age of onset of 32.2 years [1]. It is attributed to several local orbital and systemic factors but the occurrence of this condition in a patient with systemic sclerosis (SSc) is worth reporting. The impaired near vision or photophobia due to the same may hamper routine social and professional activities in the young.

## 2. Case Presentation

A 33-year-old woman, undergoing treatment for systemic sclerosis, presented to the Ophthalmology Outpatient Department with complaints of decreased near vision for the last six months. There was no history of migraine, facial or extremity weakness, dysarthria, or ataxia. There was no recent history of syphilis, viral infection, alcoholism, or malignant lymphoma.

**Figure 1:**
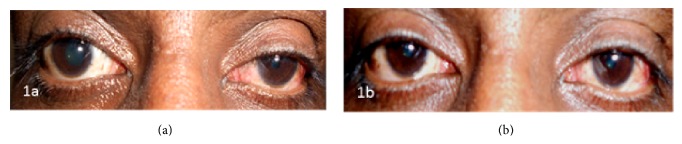
(a) Right sided irregularly dilated pupil with minimal reaction to light suggestive of tonic pupil and a normally reacting left pupil. (b) Right pupil shows constriction (denervation supersensitivity) and no constriction in left pupil with 0.125% pilocarpine.

On examination, she had a vision of 20/20 OU on the Snellen chart. However, she had a near vision of N12 in the right eye and N6 in the left eye. Slit lamp examination was normal except for the presence of anisocoria and vermiform contractions in the right eye. Her right pupil was 4.5 mm, ovally distorted, and sluggishly reacting to light and left pupil was reactive from 3 mm to 2 mm (Figure 1(a)). Pupillary reactions to accommodation were, however, normal. No abnormalities were detected in extraocular movements or fundoscopic examination. Corneal sensations were normal. The diagnosis of Adie's tonic pupil was confirmed with a rapid miotic response of the right pupil to 0.125% pilocarpine drops (Figure 1(b)).

**Figure 2:**
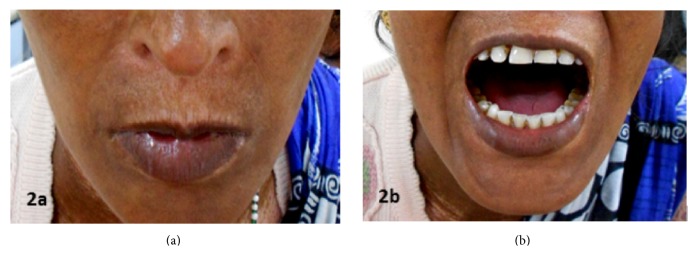
(a, b) Reduced oral aperture and resultant microstomia with limited oral access.

**Figure 3:**
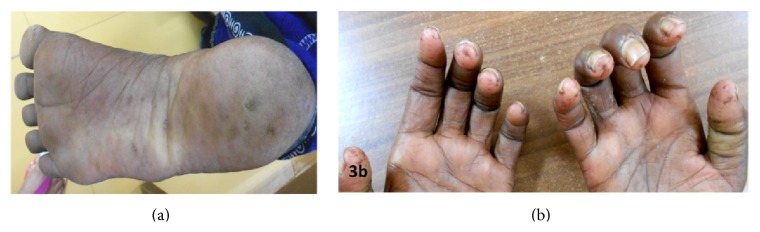
(a) Callus formation and calcinosis on left sole. (b) Digital ulceration and scarring.

On physical examination, she had mottled pigmentation with binding down of skin, reduced oral aperture, and resultant microstomia with limited oral access (Figures 2(a) and 2(b)). She also had digital ulceration and scarring on her fingertips, callus formation, and calcinosis on her left sole (Figures 3(a) and 3(b)). She complained of breathlessness occasionally; however, her computed tomography scan of thorax was within normal limits. Her deep tendon stretch reflexes were within normal limits. The patient had an unremarkable brain computed tomography (CT) and magnetic resonance imaging (MRI). Her routine hemogram was within normal limits. Echocardiography revealed grade 2 diastolic dysfunction, and nerve conduction velocities were within normal limits. Her serum antinuclear antibody HEp-2 by indirect fluorescent antibody technique was positive with a primary dilution titre of 1 : 40 and end-point titre of 1 : 640.

The need of a hyperopic correction for near vision was explained to the patient and she was prescribed +1D for the right eye. She was advised to avoid cold weather protective clothing to prevent Raynaud phenomenon. At 6-month follow-up, Adie's tonic pupil persisted. The patient was on oral corticosteroids and was systemically stable.

## 3. Discussion

Systemic sclerosis is a rare connective tissue disorder that is characterized by abnormal fibroblast proliferation leading to deposition of extracellular matrix in the skin, blood vessels, and viscera. This results in stiffening of the connective tissue structures in the skin and body organs. Like other autoimmune diseases, it is characterized by interplay of inflammatory cytokines and antibodies [2]. It is more common in women with female to male ratio being 8 : 1 [3]. Autonomic dysfunction in systemic sclerosis with both sympathetic and parasympathetic damage is also a known fact [4–8].

Most commonly reported ocular pathologies in SSc include eyelid stiffness that is seen in more than 50% of the cases and results from deposition of type I collagen in the dermis. Keratoconjunctivitis sicca is the second most common problem seen in 50% of the affected patients. Cases of conjunctivitis, episcleritis, anterior uveitis, and hypertensive retinopathy have also been reported [9–12]. Peyman et al. [13] quote that retinopathy in scleroderma is rare, but occasionally cotton wool spots, disc oedema, retinal oedema, exudates, and haemorrhages have been described. These changes may be seen in the presence or absence of hypertension.

West and Barnett [14] had reported ocular findings such as lens opacities, vitreous frosting, arteriosclerotic changes, eyelid abnormalities (stiffness or tightness in, telangiectasia), deficient tear secretion, and conjunctival abnormalities (injection and vascular sludging) in thirty-eight patients with systemic sclerosis. Anand [15] has described a patient with eyelid tightness and colloid bodies in retina.

Aynaci et al. [16] described a child with hemifacial atrophy features without any neurological deficiency, with Adie's syndrome on the affected side of the face—with mydriasis, no reaction to light, and with a slow reaction to convergence and accommodation (tonic pupil). Schnitzler et al. [17] have reported a 25-year-old patient who at the age of 14 had a tonic pupil on the right side and epilepsy attacks. Both these patients had Adie's syndrome in which Adie's tonic pupil is a part of the immunological etiology.

From the above-reported case reports we found out that the mode of clinical presentation in our patient was atypical in the sense that she presented with pupillary dysfunction as the sole ophthalmic manifestation.

Most cases of Adie's tonic pupil are idiopathic, occur in young women, and present with complaints of decreased near vision, photophobia, or asymmetric pupils [1]. A tonic pupil can occur due to local causes involving the orbit, including orbital or choroidal tumors, surgical or traumatic insult, and infection or inflammation. This disorder has also been associated with autoimmune diseases (Sjogren's syndrome and Systemic Lupus Erythematosus), neurosyphilis, diabetes, herpes zoster, giant cell arteritis, and alcoholism [1]. It is characterized by a unilateral, sometimes bilateral (10%), large, and ovally distorted pupil with decreased response to light but preserved or enhanced constriction to near vision, segmental palsy of the iris sphincter resulting in segmental iris constriction, vermiform movements of the pupillary border, and hypersensitivity to 0.125% pilocarpine drops due to denervation hypersensitivity [1].

Adie's tonic pupil as an idiopathic variety is not a rare entity and may occur independently. Having excluded all the possible aetiological factors of Adie's tonic pupil in our patient, we postulate that the occurrence of this finding in SSc is due to the autonomic dysfunction of the parasympathetic ciliary ganglion. However, there were no associated symptoms related to autonomic dysfunction like abnormal cardiac activity except for mild diastolic dysfunction and gastrointestinal motility disturbances. Hence, a hyperopic correction is usually given for correction of defective near vision and sunglasses for photophobia. Resolution of Adie's tonic pupil usually occurs within a year if isolated ciliary ganglion dysfunction is present; however, there is small but definite tendency to become worse over the years [1]. Further study in ocular manifestations in systemic sclerosis is required to validate our observation in a larger cohort.
